# Prevalence and associated factors of cognitive frailty in older adults with heart failure: a systematic review and meta-analysis

**DOI:** 10.3389/fpubh.2026.1866159

**Published:** 2026-08-11

**Authors:** Min Cheng, Jing Zhao, Juan Hu

**Affiliations:** 1 Department of Emergency Nursing, West China Second University Hospital, Sichuan University, Chengdu, China; 2 Key Laboratory of Birth Defects and Related Diseases of Women and Children (Sichuan University), Ministry of Education, Chengdu, China

**Keywords:** cognitive frailty, heart failure, meta-analysis, older adults, prevalence, related factors, systematic review

## Abstract

**Objectives:**

To explore the prevalence and associated factors of cognitive frailty in older adults with heart failure.

**Design:**

A systematic review and meta-analysis.

**Methods:**

We systematically searched China National Knowledge Infrastructure (CNKI), Wanfang Data, the VIP Database, SinoMed, PubMed, Web of Science, Embase, the Cochrane Library and Scopus from their inception to March 30, 2026.

**Results:**

A total of 22 studies involving 8,892 older adults with heart failure were included. The meta-analysis showed that the pooled prevalence of cognitive frailty in older adults with heart failure was 32% (95% CI: 27%-37%). Descriptive stratified analyses yielded pooled prevalence estimates of 37% in women and 31% in men. According to New York Heart Association functional classification, the corresponding estimates were 44% among patients classified as class IV and 28% among those classified as classes II-III.The prevalence was also higher in patients with comorbid chronic obstructive pulmonary disease (46%) and coronary heart disease (31%). Older age was associated with higher odds of cognitive frailty (adjusted OR = 1.12, 95% CI 1.06–1.18). Depression was also associated with higher odds, whereas exercise and intellectual activity were associated with lower odds.

**Conclusions and Implications:**

Cognitive frailty appears to be common among older adults with heart failure, although prevalence estimates vary across populations and assessment approaches. Older age and depression were associated with higher odds of cognitive frailty, whereas exercise and intellectual activity were associated with lower odds. These findings support early screening and comprehensive assessment, while the observed associations should not be interpreted as causal because the available evidence was predominantly observational and cross-sectional.

**Systematic Review Registration:**

https://www.crd.york.ac.uk/PROSPERO/view/CRD420261360024, PROSPERO, identifier CRD420261360024.

## 1 Introduction

Heart failure is a major clinical syndrome in later life and remains an important cause of morbidity, mortality, and hospitalization among older adults (1). Contemporary heart failure care increasingly recognizes that management in older patients extends beyond cardiac dysfunction alone, because geriatric syndromes such as frailty and cognitive impairment frequently coexist and may complicate treatment, self-management, and prognosis. Frailty and cognitive impairment are both common in patients with heart failure (2, 3). A previous meta-analysis estimated that frailty affects 38% of patients with heart failure (4), whereas a systematic review and meta-analysis reported a pooled prevalence of cognitive impairment of 43% in this population (5). Moreover, Wleklik et al. (6) reported that greater frailty was associated with poorer cognitive function in patients with heart failure. Taken together, these findings suggest that the coexistence of physical vulnerability and cognitive decline may represent a clinically meaningful phenotype in older adults with heart failure.

The concept of cognitive frailty was introduced by the International Academy on Nutrition and Aging and the International Association of Gerontology and Geriatrics and is generally defined as the coexistence of physical frailty and cognitive impairment in the absence of dementia (7). This construct has attracted growing attention because it may identify a subgroup of older adults with compounded vulnerability and elevated risk of adverse outcomes. In older patients with heart failure, the FRAGILE-heart failure study reported that 23% had cognitive frailty and that cognitive frailty was associated with a 1.55-fold higher risk of the composite outcome of all-cause mortality and heart failure rehospitalization within 1 year (8). More broadly, meta-analytic evidence in older adults has shown that cognitive frailty is associated with falls, disability, and hospitalization (9).

However, the reported prevalence of cognitive frailty in older adults with heart failure varies across studies, and the associated factors identified to date remain inconsistent. Differences in study setting, patient characteristics, heart failure severity, and the instruments used to assess frailty and cognition may partly explain these discrepancies. A clearer synthesis of the available evidence is therefore needed. Accordingly, this systematic review and meta-analysis aimed to estimate the pooled prevalence of cognitive frailty and summarize its associated factors in older adults with heart failure.

## 2 Methods

### 2.1 Design

This study was conducted in accordance with the Preferred Reporting Items for Systematic Reviews and Meta-Analyses (PRISMA 2020) statement (10). The review protocol was registered in the International Prospective Register of Systematic Reviews (PROSPERO) (CRD420261360024).

### 2.2 Search strategy

We systematically searched China National Knowledge Infrastructure (CNKI), Wanfang Data, the VIP Database, SinoMed, PubMed, Web of Science, Embase, the Cochrane Library, and Scopus from their inception to March 30, 2026. For CNKI, Wanfang Data, and the VIP Database, both journal article collections and degree thesis collections were searched. The search strategy combined subject headings and free-text terms, including “aged,” “elderly,” “cognition,” “cognitive function,” “cardiac failure,” “heart decompensation” and related terms. The search strategy is presented as an example in Supplemental Text S1.

### 2.3 Inclusion and exclusion criteria

Studies were included if they met the following criteria: (1) participants were older adults with heart failure, with a mean or median age of 60 years or older, or the study explicitly defined the population as older adults. (2) Heart failure was clearly diagnosed according to recognized clinical criteria, guidelines, medical records, or physician diagnosis. (3) Cognitive frailty was defined as the coexistence of physical frailty and cognitive impairment and/or subjective cognitive decline, with dementia excluded. (4) The study reported the prevalence of cognitive frailty and/or factors associated with cognitive frailty. (5) The study used an observational design, including a cross-sectional, case-control, cohort, or longitudinal design. (6) The publication was either a full-text journal article or a formally completed master's or doctoral thesis publicly available through a recognized academic database or institutional repository. Thesis-type records were eligible only if they reported original observational data, provided sufficient methodological information and extractable outcome data, and met all other eligibility criteria. (7) Cognitive frailty was operationally defined as the concurrent presence of physical frailty and objectively assessed cognitive impairment in individuals without diagnosed dementia. Physical frailty and cognitive impairment were identified according to the instruments and cut-off values specified in each original study. Studies were eligible only when the physical frailty and cognitive impairment components could be identified separately and their coexistence could be used to determine cognitive frailty. No restrictions were imposed on the specific assessment instruments, provided that the instruments, cut-off values, and classification procedures were clearly reported or could be obtained from the full text. For multiple reports derived from the same or potentially overlapping cohort, the authors, institutions, recruitment settings and periods, sample sizes, participant characteristics, and assessment instruments were compared. Only the report providing the most complete data relevant to the review outcomes or, where applicable, the longest follow-up was retained.

Studies were excluded if they met any of the following criteria: (1) the population did not specifically include older adults with heart failure. (2) Cognitive frailty could not be identified separately or was not clearly defined. (3) Dementia had to be excluded through an explicit study eligibility criterion, a documented clinical diagnosis, medical records, recognized diagnostic criteria, or an assessment procedure described by the original investigators. The specific method used to exclude dementia was recorded for each study. When the original study stated that dementia was excluded but did not describe the procedure, the exclusion method was classified as not clearly reported. (4) Extractable data on the prevalence or associated factors of cognitive frailty were unavailable. (5) The publication was a review, meta-analysis, conference abstract, case report, comment, editorial, protocol, letter, or an incomplete, non-final, or non-publicly available thesis or dissertation. (6) The full text was unavailable or the publication language was neither English nor Chinese.

### 2.4 Study selection and data extraction

Two reviewers independently conducted the literature search, study selection, and data extraction. Titles and abstracts were screened first, and studies that clearly did not meet the eligibility criteria were excluded. The full texts of the remaining articles were then reviewed for final inclusion. Any disagreements were resolved by discussion with a third reviewer. Extracted data included the first author, publication year, country, participants' age, study design, investigation or follow-up period, sample size, proportion of female participants, prevalence of cognitive frailty, diagnostic criteria or assessment tools for cognitive frailty, associated factors, and quality assessment scores.

### 2.5 Quality assessment

Cross-sectional studies were appraised using the 11-item Agency for Healthcare Research and Quality (AHRQ) checklist (11). Each item was rated as “yes,” “no,” or “unclear” with 1 point assigned for each “yes” response. Total scores of 8–11 indicated high quality, 4–7 moderate quality, and < 4 low quality. Cohort and longitudinal studies were assessed using the Newcastle-Ottawa Scale (NOS) (12), which evaluates study quality across 3 domains: selection, comparability, and outcome. The NOS has a maximum score of 9 points; studies scoring 7–9 were considered high quality, 4–6 moderate quality, and < 4 low quality. Quality assessment was conducted independently by 2 reviewers, and disagreements were resolved through discussion with a third reviewer.

### 2.6 Statistical analysis

The outcomes of interest were the prevalence of cognitive frailty and its associated factors. Pooled prevalence estimates and pooled odds ratios (ORs) with 95% confidence intervals (CIs) were calculated. For the analysis of associated factors, adjusted odds ratios and their 95% CIs derived from multivariable regression models were preferentially extracted. All statistical analyses were performed using Stata version 15.1. Statistical heterogeneity across studies was assessed using Cochran's *Q* test and the *I*^2^ statistic. A fixed-effects model was used when heterogeneity was low (*I*^2^ < 50%); otherwise, a random-effects model was applied. Potential sources of heterogeneity were further explored through subgroup analyses. Sensitivity analysis was conducted by sequentially omitting 1 study at a time to evaluate the robustness of the pooled results. Publication bias was assessed visually using funnel plots and statistically using Begg's and Egger's tests.

## 3 Results

**Figure 1:**
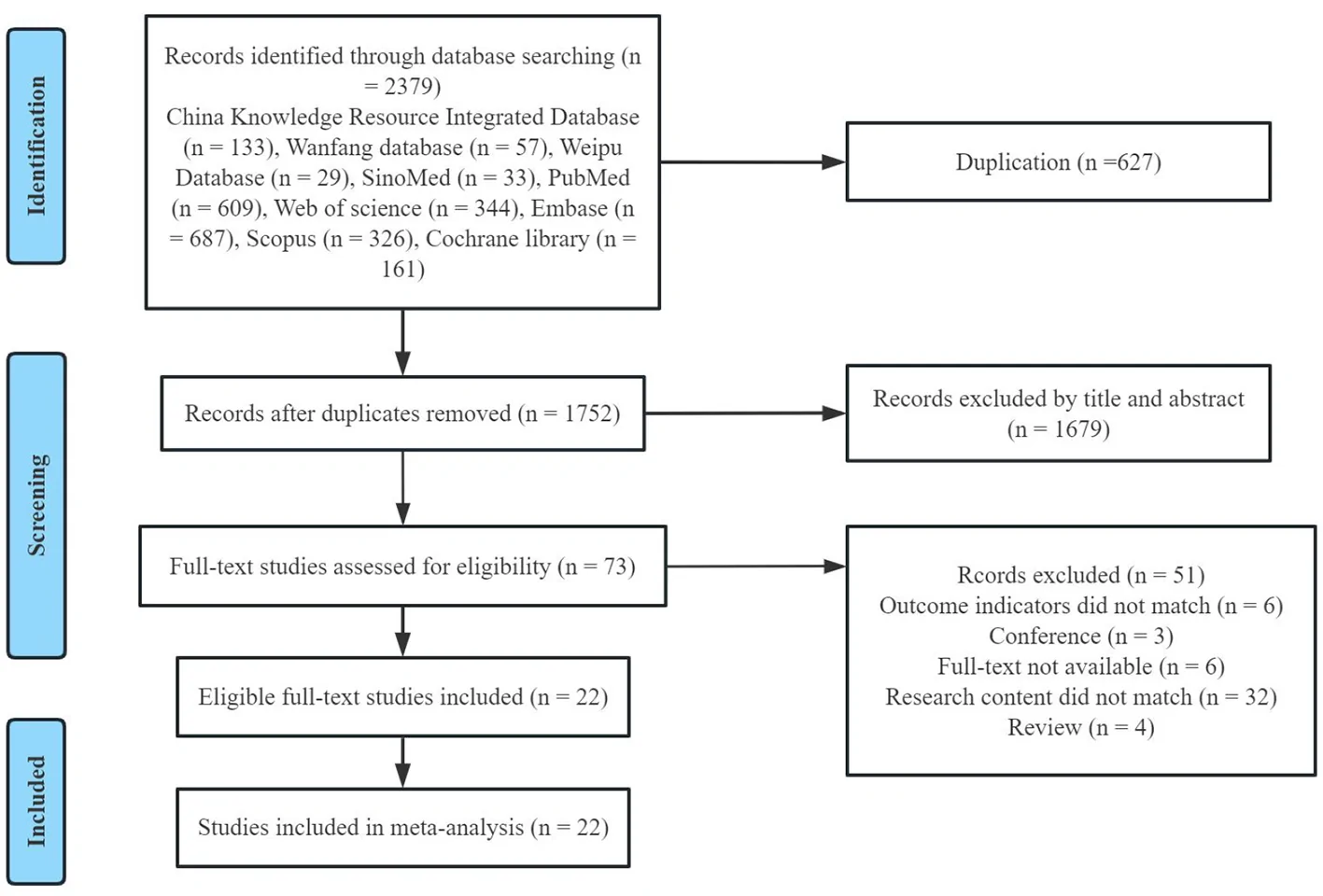
Flow diagram of study selection.

A total of 2,379 records were initially identified. After removal of 627 duplicates, 1,752 records remained for title and abstract screening. Of these, 1,679 were excluded, and 73 articles underwent full-text review. After full-text assessment, 51 articles were excluded, leaving 22 studies for inclusion in the final analysis. The study selection process is shown in Figure 1.

### 3.1 Study characteristics and quality assessment

A total of 22 studies (13–34) involving 8,892 participants were included in this review. Of the 22 included studies, 16 were journal articles and 6 were formally completed degree theses. The studies were conducted in China, Japan, Korea, Spain, and Poland. Study designs comprised 16 cross-sectional studies, 4 cohort studies, 1 longitudinal study, and 1 case-control study. Across the included studies, 9 combinations of cognitive frailty assessment tools were used, including FRAIL+Montreal Cognitive Assessment (MoCA), Frailty Phenotype (FP)+Mini-Mental State Examination (MMSE), FP+MoCA, FRAIL+MMSE, Tilburg Frailty Scale (TFS)+MoCA, TFS+MMSE, FP+Mini-Cog, Comprehensive Geriatric Assessment-Frailty Index (CGA-FI)+MMSE, and Self-Reported Barber Questionnaire (SRBQ)+MMSE. Detailed study characteristics and quality assessment results are presented in Table 1. Study quality was assessed using the AHRQ checklist for cross-sectional studies and the NOS for cohort and longitudinal studies. Overall, 17 studies were rated as high quality and 5 as moderate quality. Detailed item-level assessments are presented in Supplementary Table 1 and Supplementary Table 2.

**Table 1:** Characteristics and methodological quality of the included studies.

References	Country	Age/years	Study design	Investigation time	*N* (Female %)	Prevalence %	Cognitive frailty assessment		Associated factors	Quality
							Frailty	Cognitive impairment		
Wang et al. (17)	China	73.09 ± 5.97	Case-control study	August 2023 to August 2025	246 (48.4)	35.6	FP	MoCA	Age, exercise, depression, intellectual activity	9/9
Li et al. (27)	China	≥60	Longitudinal study	July 2022 to February 2024	622 (-)	30.7	FRAIL	MMSE	Age, exercise, depression	8/9
Chen (13)	China	66 (62, 71)	Cross-sectional study	September 2023 to October 2024	607 (42.2)	48.3	FP	MoCA	Age, exercise	9/11
Guo (14)	China	≥60	Cross-sectional study	December 2023 to January 2025	337 (-)	44.5	TFS	MoCA	Age	7/11
Wang et al. (15)	China	≥65	Cross-sectional study	January to October in 2021	124 (50.0)	29.03	FP	MMSE	Age	10/11
Wang et al. (16)	China	≥60	Cross-sectional study	June to October 2021	590 (47.8)	33.6	TFS	MMSE	-	8/11
Luo (20)	China	≥60	Cross-sectional study	May 2022 to December 2023	451 (-)	31.9	FP	MMSE	Age, exercise, depression	9/11
Jiang (18)	China	≥60	Cross-sectional study	May 2021 to September 2022	393 (52.7)	34.9	FP	MoCA	Age, depression, intellectual activity	8/11
Liu (19)	China	70.2 ± 6.8	Cross-sectional study	August 2021 to September 2022	327 (45.3)	50.5	FRAIL	MoCA	-	9/11
Wang et al. (21)	China	76.5 ± 7.9	Cross-sectional study	November 2015 to January 2018	146 (-)	43.2	CGA-FI	MMSE	-	6/11
Jiang et al. (23)	China	≥60	Cross-sectional study	October 2023 to January 2024	330 (45.5)	37.6	FP	MoCA	Age, exercise, depression, intellectual activity	8/11
Cui (22)	China	≥60	Cross-sectional study	March 2023 to December 2023	181 (-)	56.4	FP	MoCA	-	6/11
Matsue et al. (30)	Japan	≥65	Cohort study	September 2016 to March 2018	1180 (52.6)	18.2	FP	Mini-Cog	-	8/9
Gou et al. (25)	China	73.00 ± 6.95	Cross-sectional study	August 2021 and November 2022	421 (51.8)	19.7	FRAIL	MMSE	Age, depression	9/11
Seo and Son (31)	Korea	72.40 ± 5.94	Cross-sectional study	December 2016 to April 2017	168 (26.8)	34.5	FRAIL	MMSE	-	9/11
Xu et al. (34)	China	≥60	Cross-sectional study	August 2022 to February 2023	207 (-)	35.3	FRAIL	MoCA	-	6/11
Seo et al. (32)	Korea	≥65	Cross-sectional study	February to August in 2018	176 (43.2)	14.2	FRAIL	MMSE	-	7/11
González-Moneo et al. (24)	Spain	71 ± 11	Cohort study	2005 to 2010	525 (39.0)	17.7	SRBQ	MMSE	-	8/9
Wleklik et al. (33)	Poland	72.32 ± 6.73	Cohort study	September 2022 and June 2023	250 (30.8)	18.4	FP	MMSE	-	8/9
Liu et al. (28)	China	66.9 ± 6.4	Cohort study	September 2023 to December 2024	334 (40.7)	31.7	FRAIL	MoCA	-	8/9
Liu et al. (29)	China	69.97 ± 6.5	Cross-sectional study	October 2022 to August 2023	470 (43.0)	44.5	FRAIL	MoCA	Age	9/11
Gou et al. (26)	China	60–93	Cross-sectional study	April 2023 to May 2024	807 (51.8)	17.8	FRAIL	MoCA	-	9/11

FP, Frailty Phenotype; MMSE, Mini-Mental Status Examination; MoCA, Montreal Cognitive Assessment; CGA-FI, Comprehensive Geriatric Assessment-Frailty Index; TFS, Tilburg Frailty Scale; SRBQ, Self-Reported Barber Questionnaire; -, not applicable.

### 3.2 Prevalence of cognitive frailty in older adults with heart failure

**Figure 2:**
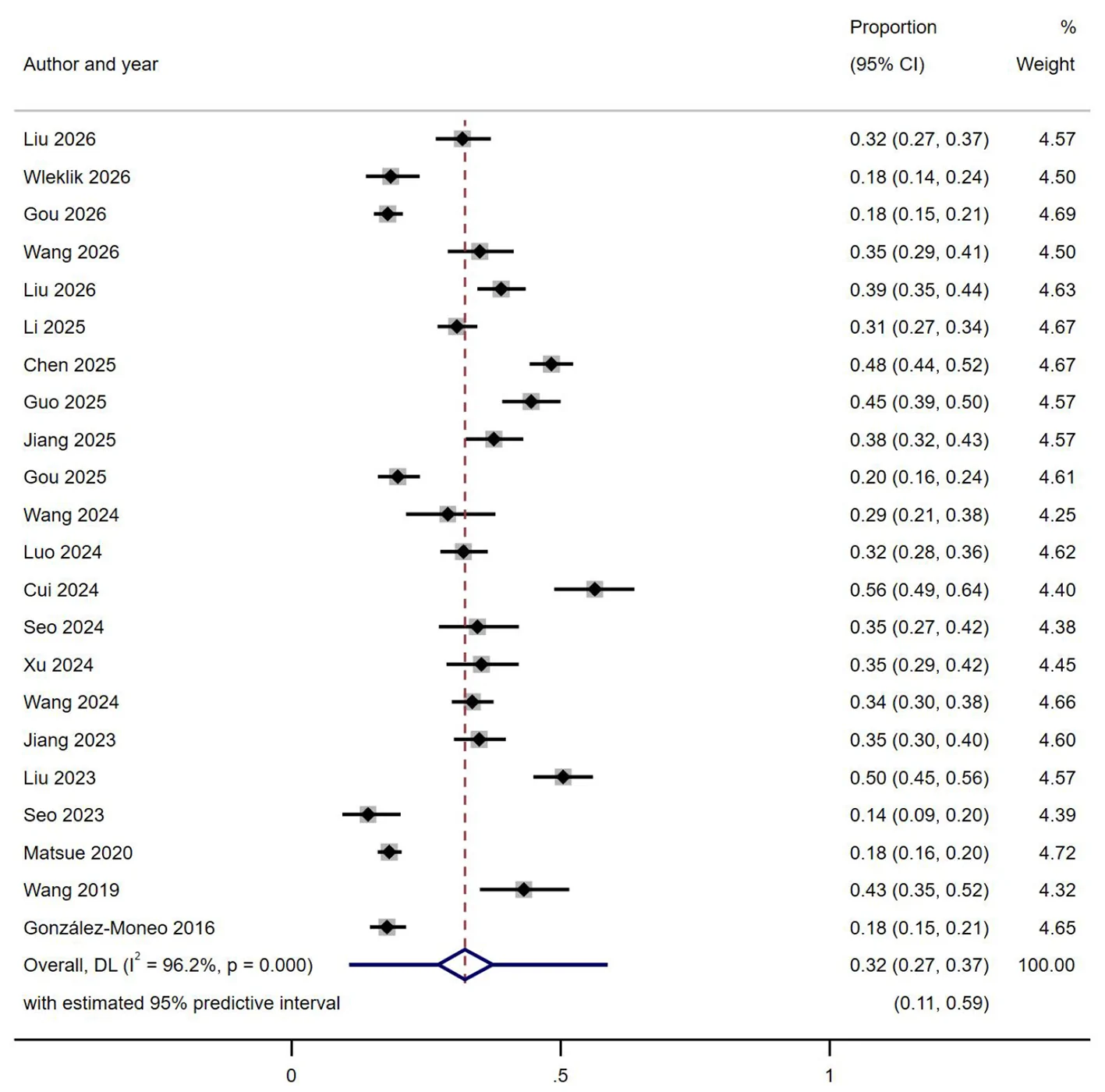
Forest plot of the prevalence of cognitive frailty among older adults with heart failure.

A random-effects model was used because of the substantial between-study heterogeneity (*I*^2^= 96.2%, *Tau*^2^= 0.06, *p* < 0.01). The pooled prevalence of cognitive frailty among older adults with heart failure was 32% (95% CI: 27%−37%; 95% prediction interval: 11%−59%) (Figure 2). Subgroup analyses were conducted according to 11 factors: sex, educational level, marital status, NYHA functional class, comorbidities, cognitive frailty assessment tools, study design, smoking, drinking, sleep disorders, and malnutrition. Descriptive stratified analyses showed pooled prevalence estimates of 37% (95% CI 30%−43%) among women and 31% (95% CI 23%−39%) among men; 46% (95% CI 37%−56%) among participants with primary school education or below and 25% (95% CI 18%−33%) among those with middle school education or above; 49% (95% CI 35%−64%) among unmarried or other-status participants and 29% (95% CI 22%−35%) among married participants; and 44% (95% CI 32%−56%) among patients with NYHA class IV heart failure and 28% (95% CI 21%−36%) among those with NYHA class II-III heart failure. Because these estimates were pooled separately and no formal between-group comparisons were conducted, the observed differences should be interpreted as descriptive patterns only. In the subgroup analyses according to comorbidities, the pooled prevalence of cognitive frailty was 43% (95% CI: 36%−50%) among patients with diabetes, 32% (95% CI: 24%−41%) among those with hypertension, 46% (95% CI: 29%−63%) among those with chronic obstructive pulmonary disease, 31% (95% CI: 22%−41%) among those with coronary heart disease, and 37% (95% CI: 29%−45%) among those with chronic renal failure. The pooled prevalence also varied according to the cognitive frailty assessment tool used, ranging from 18% to 45%. Specifically, the prevalence was 34% (95% CI: 23%−47%) for FRAIL combined with MoCA, 26% (95% CI: 18%−36%) for the FP combined with MMSE, 42% (95% CI: 35%−50%) for FP combined with MoCA, and 24% (95% CI: 16%−33%) for FRAIL combined with MMSE. The corresponding prevalence was 45% (95% CI: 39%−50%) for the TFS combined with MoCA, 34% (95% CI: 30%−38%) for TFS combined with MMSE, 18% (95% CI: 16%−20%) for FP combined with the Mini-Cog, 43% (95% CI: 35%−52%) for the CGA-FI combined with MMSE, and 18% (95% CI: 15%−21%) for the SRBQ combined with MMSE. According to study design, the pooled prevalence was 21% (95% CI: 16%−27%) in cohort studies, 35% (95% CI: 29%−41%) in cross-sectional studies, 31% (95% CI: 27%−34%) in longitudinal studies, and 35% (95% CI: 29%−41%) in case-control studies. In addition, the pooled prevalence of cognitive frailty was 28% (95% CI: 21%−36%) among smokers, 29% (95% CI: 22%−38%) among drinkers, 35% (95% CI: 27%-44%) among participants with sleep disorders, and 38% (95% CI: 28%−49%) among those with malnutrition. The detailed results of the subgroup analyses are presented in Table 2.

**Table 2:** Stratified pooled prevalence estimates of cognitive frailty in older adults with heart failure.

Subgroup	Number of studies	*I*^2^ (%)	Pooled prevalence (95% CI)	*p*
Gender				
Female	13	89.04	0.37 (0.30, 0.43)	< 0.01
Male	13	94.78	0.31 (0.23, 0.39)	< 0.01
Education				
Primary school and below	12	95.48	0.46 (0.37, 0.56)	< 0.01
Middle school level and above	12	93.87	0.25 (0.18, 0.33)	< 0.01
Marriage				
Married	11	94.83	0.29 (0.22, 0.35)	< 0.01
Unmarried or otherwise	11	94.91	0.49 (0.35, 0.64)	< 0.01
Cardiac function classification				
II-III	11	95.18	0.28 (0.21, 0.36)	< 0.01
IV	9	91.53	0.44 (0.32, 0.56)	< 0.01
Combining chronic diseases				
Diabetes	7	56.67	0.43 (0.36, 0.50)	< 0.01
Hypertension	9	88.38	0.32 (0.24, 0.41)	< 0.01
COPD	3	47.80	0.46 (0.29, 0.63)	< 0.01
Coronary heart disease	8	93.52	0.31 (0.22, 0.41)	< 0.01
Chronic renal failure	3	0.00	0.37 (0.29, 0.45)	< 0.01
Cognitive frailty assessment tool				
FRAIL+MoCA	5	97.21	0.34 (0.23, 0.47)	< 0.01
FP+MMSE	3	-	0.26 (0.18, 0.36)	< 0.01
FP+MoCA	5	90.19	0.42 (0.35, 0.50)	< 0.01
FRAIL+MMSE	4	91.87	0.24 (0.16, 0.33)	< 0.01
TFS+MoCA	1	-	0.45 (0.39, 0.50)	< 0.01
TFS+MMSE	1	-	0.34 (0.30, 0.38)	< 0.01
FP+Mini-Cog	1	-	0.18 (0.16, 0.20)	< 0.01
CGA-FI+MMSE	1	-	0.43 (0.35, 0.52)	< 0.01
SRBQ+MMSE	1	-	0.18 (0.15, 0.21)	< 0.01
Study type				
Cohort study	4	89.71	0.21 (0.16, 0.27)	< 0.01
Cross-sectional study	16	95.71	0.35 (0.29, 0.41)	< 0.01
Longitudinal study	1	-	0.31 (0.27,0.34)	< 0.01
Case-control study	1	-	0.35 (0.29, 0.41)	< 0.01
Smoking	11	86.34	0.28 (0.21, 0.36)	< 0.01
Drinking	11	86.32	0.29 (0.22, 0.38)	< 0.01
Sleep disorders	9	92.50	0.35 (0.27, 0.44)	< 0.01
Malnutrition	8	92.59	0.38 (0.28, 0.49)	< 0.01

FP, Frailty Phenotype; MMSE, Mini-Mental Status Examination; MoCA, Montreal Cognitive Assessment; CGA-FI, Comprehensive Geriatric Assessment-Frailty Index; TFS, Tilburg Frailty Scale; SRBQ, Self-Reported Barber Questionnaire; COPD, Chronic Obstructive Pulmonary Disease; -, not applicable.

### 3.3 Risk factor cognitive frailty among older adults with heart failure

#### 3.3.1 Age

Ten studies reported the association between age and cognitive frailty in older adults with heart failure. A random-effects model was used for the meta-analysis because of substantial heterogeneity (*I*^2^ = 60.6%, *p* = 0.007). The pooled results showed that older age was associated with higher odds of cognitive frailty. The pooled adjusted odds ratio was 1.12 (95% CI 1.06–1.18) per 1-year increase in age (Table 3).

**Table 3:** Associated factors of cognitive frailty in older adults with heart failure.

Risk factor	Number of studies	Heterogeneity		Meta-analysis	
		*I*^2^ (%)	*p*	OR (95% CI)	*p*
Age	10	60.6	< 0.007	1.12 (1.06, 1.18)	< 0.001
Exercise	5	0.00	0.481	0.22 (0.15, 0.32)	< 0.001
Depression	6	41.5	0.128	4.42 (3.28, 5.95)	< 0.001
Intellectual activity	3	69.7	0.037	0.22 (0.10, 0.50)	< 0.001

#### 3.3.2 Exercise

Five studies reported the association between exercise and cognitive frailty. Given the low heterogeneity among studies (*I*^2^ = 0.0%, *p* = 0.481), a fixed-effects model was used for the meta-analysis. The pooled results showed that engagement in exercise was associated with lower odds of cognitive frailty (OR = 0.22, 95% CI 0.15–0.32) (Table 3).

#### 3.3.3 Depression

Six studies reported the association between depression and cognitive frailty. Given the moderate heterogeneity among studies (*I*^2^ = 41.5%, *p* = 0.128), a fixed-effects model was used for the meta-analysis. The pooled results showed that depression was associated with higher odds of cognitive frailty (OR = 4.42, 95% CI 3.28–5.95) (Table 3).

#### 3.3.4 Intellectual activity

A total of 3 studies described the relationship between intellectual activity and cognitive frailty. Given the substantial heterogeneity among studies (*I*^2^ = 69.7%, *p* = 0.037), a random-effects model was used for the meta-analysis. The results indicated that engagement in intellectual activity was associated with lower odds of cognitive frailty in older adults with heart failure (OR = 0.22, 95% CI 0.10–0.50) (Table 3).

### 3.4 Sensitivity analysis

**Figure 3:**
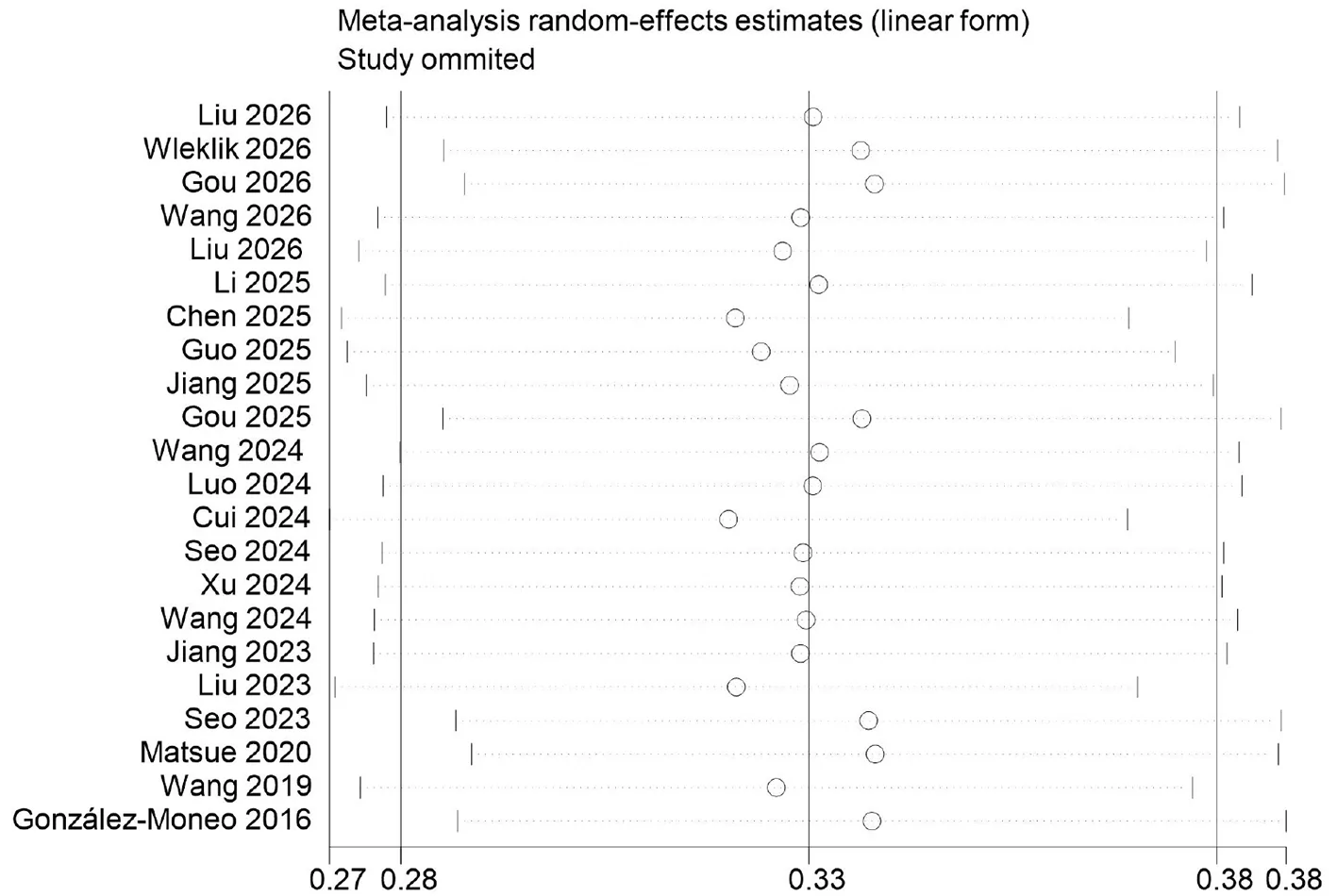
Leave-one-out sensitivity analysis of the pooled prevalence of cognitive frailty among older adults with heart failure.

Sensitivity analysis was performed for the pooled prevalence. Sequential omission of individual studies did not materially alter the results, indicating that the pooled estimate derived from the random-effects model was robust. The results of the sensitivity analysis are presented in Figure 3.

### 3.5 Publication bias

**Figure 4:**
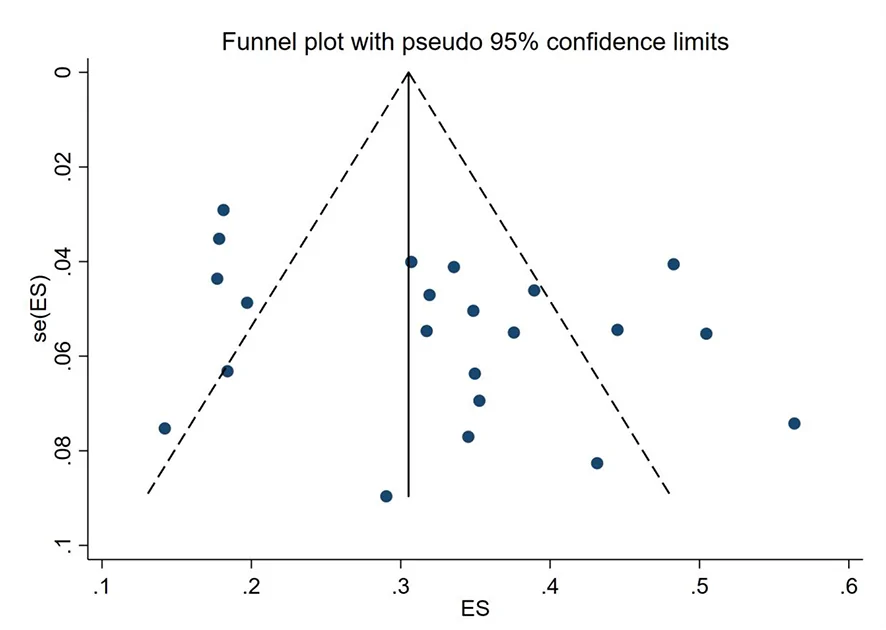
Funnel plot of the prevalence of cognitive frailty in older adults with heart failure.

Potential small-study effects and publication bias were explored for the pooled prevalence using a funnel plot, Egger's test, and Begg's test. Visual inspection of the funnel plot did not reveal marked asymmetry. Neither Egger's test (*p* = 0.074) nor Begg's test (*p* = 0.310) indicated statistically significant funnel-plot asymmetry. However, given the substantial between-study heterogeneity and the limited interpretability of funnel plots and asymmetry tests in prevalence meta-analyses, these findings should be interpreted cautiously and do not exclude the possibility of publication bias, selective reporting, or other small-study effects. The funnel plot is presented in Figure 4.

## 4 Discussion

This systematic review and meta-analysis included 22 studies and summarized the prevalence of cognitive frailty and its associated factors among older adults with heart failure. The pooled prevalence of cognitive frailty in this population was 32%. In addition, older age and depression were associated with higher odds of cognitive frailty, whereas exercise and intellectual activity were associated with lower odds of cognitive frailty.

A recent meta-analysis including 90 studies reported a pooled prevalence of 5% for cognitive frailty among older adults (35). In addition, Zheng et al. (36) found that the prevalence of cognitive frailty was 26.3% in a cross-sectional study of 304 older patients with lung cancer undergoing chemotherapy. Both estimates were lower than the 32% observed in our meta-analysis. This difference may be explained by the particularly high vulnerability of older adults with heart failure, in whom physical frailty and cognitive impairment often coexist. Heart failure has been linked to cognitive dysfunction through several mechanisms, including reduced cardiac output, chronic cerebral hypoperfusion, vascular pathology, systemic inflammation, and neurohormonal activation (37). At the same time, heart failure is frequently accompanied by sarcopenia, reduced exercise capacity, and impaired physiologic reserve, all of which may promote the development of frailty (2). In addition, older patients with heart failure often have a high burden of comorbidities and functional impairment, which may further increase their susceptibility to cognitive frailty (38). Therefore, the relatively high prevalence found in our study is biologically plausible and underscores the importance of early screening and multidomain intervention in this population.

Subgroup analyses provided further insight into the distribution of cognitive frailty among older adults with heart failure. The pooled prevalence estimate was numerically greater among women than among men. This pattern may reflect a combination of biological vulnerability and psychosocial factors. Women with heart failure have been reported to experience a greater burden of physical frailty than men, which may increase their susceptibility to cognitive frailty, a condition characterized by the coexistence of physical frailty and cognitive impairment (39). In addition, the decline in estrogen levels after menopause may reduce cardiovascular and neurocognitive protection, thereby increasing vulnerability to frailty and cognitive decline in later life (40). Nevertheless, this sex-related pattern should be interpreted cautiously because of heterogeneity in study populations, assessment instruments, and diagnostic thresholds (41). Descriptive stratified analyses also yielded a pooled prevalence estimate of 46% among participants with a primary school education or below, compared with 25% among those with a middle school education or above. One possible explanation is that lower educational attainment may be associated with reduced cognitive reserve. Among patients with heart failure, lower educational attainment has been identified as an important correlate of cognitive impairment (42). One study involving older adults with heart failure found that a lower educational level was associated with poorer performance on the Mini-Mental State Examination and a greater prevalence of mild cognitive impairment (42). Previous research has also suggested that education may buffer the adverse effects of cardiovascular risk factors on late-life cognition, implying that individuals with higher educational attainment may be more resilient to cardiovascular burden and age-related brain changes (43). Regarding marital status, the pooled prevalence estimate was 49% among unmarried participants or those in other marital-status categories and 29% among married participants. Marital relationships may provide relatively stable informal social support. A study of Chinese older adults found that individuals with a spouse tended to receive greater informal support and had a lower risk of cognitive impairment, whereas those without a spouse generally had less support and poorer cognitive function (44). Social isolation and loneliness may also be relevant, as both have been independently associated with cognitive frailty among older adults after adjustment for potential confounders (45). According to New York Heart Association functional classification, the pooled prevalence estimate was 44% among patients with class IV heart failure and 28% among those with classes II-III heart failure. The numerically greater estimate observed in patients with class IV heart failure may be related to more severe hemodynamic impairment, a greater inflammatory burden, and poorer functional status (46). Advanced heart failure is characterized by reduced cardiac output and cerebral hypoperfusion, which may contribute to cognitive decline (46). Heightened neurohormonal activation and systemic inflammation in severe heart failure may further impair neurological function (47). However, because these subgroup-specific estimates were pooled separately and no formal between-group comparisons were performed, all observed differences should be regarded as descriptive and hypothesis-generating rather than confirmatory. In terms of comorbid chronic diseases, older patients with heart failure who had COPD showed the highest prevalence of cognitive frailty. This may be attributed to the combined effects of hypoxia, systemic inflammation, and functional impairment. COPD was characterized by chronic hypoxemia and hypercapnia, which may directly impair cerebral function (48). When combined with heart failure-related cerebral hypoperfusion, these factors may exacerbate cognitive decline (49). In addition, both COPD and heart failure were associated with systemic inflammation and neurohormonal activation, which may accelerate muscle wasting and frailty progression (50, 51). Patients with both conditions often experience markedly reduced exercise capacity, leading to sarcopenia and physical frailty.

In the subgroup analyses, a higher prevalence of cognitive frailty was observed in studies using the TFS combined with the MoCA and in observational studies. However, both findings should be interpreted with caution because each subgroup was represented by only one study. Therefore, these differences may not be robust and could reflect study-specific characteristics, sample composition, or measurement variability rather than true differences related to the assessment tool or study design. Further studies are needed to confirm these findings. In terms of lifestyle factors, smoking and drinking may play a role in the development of cognitive frailty. Smoking has been associated with vascular injury and poorer frailty outcomes in older adults (52). In addition, smoking has also been linked to reduced cerebral perfusion, brain atrophy, and biomarkers related to neurodegeneration (53). All of which may contribute to both cognitive impairment and physical frailty. Drinking also affect cognitive frailty through multiple pathways. Excessive alcohol intake can lead to direct neurotoxicity, nutritional deficiencies, and impaired liver function, all of which may negatively impact cognitive function and physical health (54). Sleep disorders and malnutrition may also contribute to the development of cognitive frailty in older patients with heart failure. Poor sleep quality may exacerbate fatigue and reduce physical activity, thereby increasing the risk of frailty (55). Ma et al. (56) also confirmed this, showing that insomnia and poor sleep quality were associated with the presence of cognitive frailty. Malnutrition was another important factor that may predispose patients to cognitive frailty. Inadequate nutritional intake has been linked to muscle loss, reduced physical function, and impaired cognitive performance (57). Nutritional deficiencies, particularly in protein and micronutrients, may contribute to sarcopenia and neurocognitive dysfunction (58).

In terms of associated factors, older age was associated with higher odds of cognitive frailty. Advancing age is associated with a higher prevalence of sarcopenia, reduced physical function, and multisystem decline, as well as higher odds of physical frailty (25). At the same time, aging itself is closely associated with cognitive decline (59). In addition, heart failure may impair cognitive function through reduced cardiac output, chronic cerebral hypoperfusion, and cerebral hypoxia, while the aging brain is less able to tolerate these hemodynamic abnormalities (60). Previous reviews have shown that cognitive impairment in patients with heart failure is associated with cerebral hypoperfusion and structural and functional brain changes (61). Therefore, among older adults with heart failure, physical frailty and cognitive impairment may be more likely to coexist, which may be associated with higher odds of cognitive frailty. Depression was associated with higher odds of cognitive frailty in older patients with heart failure. Depressive symptoms have been associated with poorer cognitive performance in this population and may also contribute to physical frailty by reducing physical activity, worsening fatigue, impairing self-care, and increasing social withdrawal (42, 62). In addition, recent evidence suggests that depression symptoms may mediate the relationship between frailty and cognitive function in older patients with heart failure (63). Regarding factors associated with lower odds, engagement in exercise was associated with lower odds of cognitive frailty among older adults with heart failure. Regular exercise may benefit both cognitive function and physical frailty by improving cerebral and systemic perfusion, reducing inflammation and oxidative stress, promoting neuroplasticity, and enhancing muscle strength and physical performance (64). In patients with heart failure, these effects may help preserve functional reserve and reduce vulnerability to both frailty and cognitive decline (65). Engagement in intellectual activity was associated with lower odds of cognitive frailty. Previous studies suggest that cognitive reserve, particularly its leisure activity component, is associated with a lower risk of cognitive frailty, and in heart failure, greater cognitive reserve may buffer the adverse association between disease severity and cognitive function (66, 67). Taken together, these findings suggest that exercise and intellectually stimulating activities may help delay the overlap between physical frailty and cognitive impairment.

The present systematic review and meta-analysis has several limitations. First, substantial heterogeneity was observed across the included studies, which may be attributable to differences in study populations, severity of cardiac dysfunction, comorbidities, sampling methods, and assessment approaches. Although most studies were classified as having moderate or high methodological quality based on their total scores, item-level assessments revealed several potential sources of bias. Many studies recruited participants from a single hospital or used convenience sampling, which may have limited sample representativeness and reduced the generalizability of the pooled prevalence estimates. In addition, considerable variation was observed in the instruments and cut-off values used to identify physical frailty and cognitive impairment, potentially resulting in clinically heterogeneous populations being classified under the same cognitive frailty construct. The procedures used to exclude dementia were also insufficiently described in some studies; therefore, the possible inclusion of participants with undiagnosed dementia cannot be completely ruled out and may have affected the estimated prevalence. Furthermore, adjustment for confounding factors varied considerably across studies, and the covariates included in multivariable regression models were inconsistent, leaving the possibility of residual confounding. Some subgroup analyses were based on a limited number of studies, with several subgroups including only one study, and these findings should therefore be interpreted cautiously. Most included studies were conducted in China, with limited evidence from other countries, which may restrict the generalizability of the pooled estimates to other healthcare systems, ethnic groups, and clinical settings. Finally, because protocols or prespecified statistical analysis plans were unavailable for most studies, selective reporting of associated factors or statistically significant findings could not be excluded, and potential publication bias remained possible despite the absence of significant asymmetry in formal tests.

## 5 Conclusion

In conclusion, cognitive frailty is common among older adults with heart failure. Older age and depression were associated with higher odds of cognitive frailty, whereas physical exercise and intellectual activity were associated with lower odds. These findings highlight the importance of early screening and comprehensive assessment of cognitive frailty in this population. Greater attention to potentially modifiable associated factors may support the identification of individuals with higher odds of cognitive frailty and inform the development of appropriate management strategies.

## Data Availability

The original contributions presented in the study are included in the article/Supplementary material, further inquiries can be directed to the corresponding authors.
