# Supplementary material for: Prevalence and associated factors of cognitive frailty in older adults with heart failure: a systematic review and meta-analysis
Source: Front Public Health. 2026 Aug 11;14:1866159. doi: 10.3389/fpubh.2026.1866159 (PMC13505406; doi:10.3389/fpubh.2026.1866159)
Supplement: Supplementary file 1 [file Supplementary_file_1.docx]

**Search strategy**

1. **PubMed**

#1 Heart Failure[MeSH]

#2 Cardiac Failure [Title/Abstract] Heart Decompensation [Title/Abstract] OR Decompensation, Heart [Title/Abstract] OR Congestive Heart Failure [Title/Abstract] OR Heart Failure, Congestive [Title/Abstract] OR Heart Failure, Right-Sided [Title/Abstract] OR Heart Failure, Right Sided [Title/Abstract] OR Right-Sided Heart Failure [Title/Abstract] OR Right Sided Heart Failure [Title/Abstract] OR Heart Failure, Left-Sided [Title/Abstract] OR Heart Failure, Left Sided [Title/Abstract] OR Left-Sided Heart Failure [Title/Abstract] OR Left Sided Heart Failure [Title/Abstract] OR Myocardial Failure [Title/Abstract]

#3 #1 OR #2

#4 Cognition [MeSH]

#5 Cognitions [Title/Abstract] OR Cognitive Function [Title/Abstract] OR Cognitive Functions [Title/Abstract] OR Function, Cognitive [Title/Abstract] OR Cognitive Frailty [Title/Abstract] OR Cognitive Impairment [Title/Abstract] OR Cognitive Decline [Title/Abstract]

#6 #4 OR #5

#7 Frailty [MeSH]

#8 Frailties [Title/Abstract] OR Frailness [Title/Abstract] OR Frailty Syndrome [Title/Abstract] OR Debility [Title/Abstract] OR Debilities [Title/Abstract] OR Frail [Title/Abstract]

#9 #7 OR #8

#7 #3 AND #6 AND #9

**2. Web of Science**

TS = ("heart failure" OR "cardiac failure" OR "myocardial failure" OR "heart decompensation" OR "cardiac decompensation" OR "congestive heart failure" OR "chronic heart failure" OR "acute heart failure") AND TS = (cognit* OR "cognitive function*" OR "cognitive impairment*" OR "cognitive dysfunction*" OR "cognitive decline" OR "cognitive deficit*" OR "cognition disorder*" OR "mild cognitive impairment" OR "subjective cognitive decline" OR "cognitive frailty" OR "cognitive frail*" OR "reversible cognitive frailty" OR "potentially reversible cognitive frailty")
AND TS = (frail* OR "physical frailty" OR "physical frail*" OR "frailty syndrome" OR "frailty phenotype" OR "frailty index" OR "cognitive frailty" OR "cognitive frail*" OR "reversible cognitive frailty" OR "potentially reversible cognitive frailty")
AND TS = (aged OR elderly OR geriatric* OR "older adult*" OR "older people" OR "older person*" OR "older patient*" OR "older population*" OR "senior adult*" OR "middle-aged and older" OR "middle aged and older")

**3. Embase**

('heart failure'/exp OR 'heart failure':ti,ab,kw OR 'cardiac failure':ti,ab,kw OR 'myocardial failure':ti,ab,kw OR 'heart decompensation':ti,ab,kw OR 'cardiac decompensation':ti,ab,kw OR 'congestive heart failure':ti,ab,kw OR 'chronic heart failure':ti,ab,kw OR 'acute heart failure':ti,ab,kw)
AND ('cognitive defect'/exp OR 'mild cognitive impairment'/exp OR cognit*:ti,ab,kw
OR 'cognitive function*':ti,ab,kw OR 'cognitive impairment*':ti,ab,kw OR 'cognitive dysfunction*':ti,ab,kw OR 'cognitive decline':ti,ab,kw OR 'cognitive deficit*':ti,ab,kw
OR 'cognition disorder*':ti,ab,kw OR 'mild cognitive impairment':ti,ab,kw OR 'subjective cognitive decline':ti,ab,kw OR 'cognitive frailty':ti,ab,kw OR 'cognitive frail*':ti,ab,kw
OR 'reversible cognitive frailty':ti,ab,kw OR 'potentially reversible cognitive frailty':ti,ab,kw)
AND ('frailty'/exp OR frail*:ti,ab,kw OR 'physical frailty':ti,ab,kw OR 'physical frail*':ti,ab,kw OR 'frailty syndrome':ti,ab,kw OR 'frailty phenotype':ti,ab,kw OR 'frailty index':ti,ab,kw OR 'cognitive frailty':ti,ab,kw OR 'cognitive frail*':ti,ab,kw OR 'reversible cognitive frailty':ti,ab,kw OR 'potentially reversible cognitive frailty':ti,ab,kw)
AND ('aged'/exp OR aged:ti,ab,kw OR elderly:ti,ab,kw OR geriatric*:ti,ab,kw OR 'older adult*':ti,ab,kw OR 'older people':ti,ab,kw OR 'older person*':ti,ab,kw OR 'older patient*':ti,ab,kw OR 'older population*':ti,ab,kw OR 'senior adult*':ti,ab,kw OR 'middle-aged and older':ti,ab,kw OR 'middle aged and older':ti,ab,kw)

**4. Cochrane Library**

#1 MeSH descriptor: [Heart Failure] explode all trees
#2 ("heart failure" OR "cardiac failure" OR "myocardial failure" OR "heart decompensation" OR "cardiac decompensation" OR "congestive heart failure" OR "chronic heart failure" OR "acute heart failure"):ti,ab,kw
#3 #1 OR #2
#4 MeSH descriptor: [Cognitive Dysfunction] explode all trees
#5 (cognit* OR "cognitive function*" OR "cognitive impairment*" OR "cognitive dysfunction*" OR "cognitive decline" OR "cognitive deficit*" OR "cognition disorder*" OR "mild cognitive impairment" OR "subjective cognitive decline" OR "cognitive frailty" OR "cognitive frail*" OR "reversible cognitive frailty" OR "potentially reversible cognitive frailty"):ti,ab,kw
#6 #4 OR #5
#7 MeSH descriptor: [Frailty] explode all trees
#8 (frail* OR "physical frailty" OR "physical frail*" OR "frailty syndrome" OR "frailty phenotype" OR "frailty index" OR "cognitive frailty" OR "cognitive frail*" OR "reversible cognitive frailty" OR "potentially reversible cognitive frailty"):ti,ab,kw
#9 #7 OR #8
#10 MeSH descriptor: [Aged] explode all trees
#11 MeSH descriptor: [Aged, 80 and over] explode all trees
#12 (aged OR elderly OR geriatric* OR "older adult*" OR "older people" OR "older person*" OR "older patient*" OR "older population*" OR "senior adult*" OR "middle-aged and older" OR "middle aged and older"):ti,ab,kw
#13 #10 OR #11 OR #12
#14 #3 AND #6 AND #9 AND #13

**5. Scopus**

TITLE-ABS-KEY("heart failure" OR "cardiac failure" OR "myocardial failure" OR "heart decompensation" OR "cardiac decompensation" OR "congestive heart failure" OR "chronic heart failure" OR "acute heart failure")
AND TITLE-ABS-KEY(cognit* OR "cognitive function*" OR "cognitive impairment*" OR "cognitive dysfunction*" OR "cognitive decline" OR "cognitive deficit*" OR "cognition disorder*" OR "mild cognitive impairment" OR "subjective cognitive decline" OR "cognitive frailty" OR "cognitive frail*" OR "reversible cognitive frailty" OR "potentially reversible cognitive frailty")
AND TITLE-ABS-KEY(frail* OR "physical frailty" OR "physical frail*" OR "frailty syndrome" OR "frailty phenotype" OR "frailty index" OR "cognitive frailty" OR "cognitive frail*" OR "reversible cognitive frailty" OR "potentially reversible cognitive frailty")
AND TITLE-ABS-KEY(aged OR elderly OR geriatric* OR "older adult*" OR "older people" OR "older person*" OR "older patient*" OR "older population*" OR "senior adult*" OR "middle-aged and older" OR "middle aged and older")

**6. China National Knowledge Infrastructure (CNKI)**

SU = ('心力衰竭' OR '心衰' OR '慢性心力衰竭' OR '充血性心力衰竭' OR '急性心力衰竭' OR '心功能不全' OR '心脏衰竭' OR '心脏失代偿')
AND SU = ('认知衰弱' OR '认知虚弱' OR '认知衰弱症' OR '可逆性认知衰弱' OR '潜在可逆性认知衰弱' OR '认知' OR '认知功能' OR '认知障碍' OR '认知功能障碍' OR '认知功能下降' OR '认知下降' OR '认知减退' OR '轻度认知障碍' OR '主观认知下降')
AND SU = ('认知衰弱' OR '认知虚弱' OR '认知衰弱症' OR '衰弱' OR '虚弱' OR '老年衰弱' OR '身体衰弱' OR '躯体衰弱' OR '生理性衰弱' OR '衰弱综合征' OR '可逆性认知衰弱' OR '潜在可逆性认知衰弱')
AND SU = ('老年' OR '老年人' OR '老年患者' OR '高龄' OR '老龄' OR '中老年' OR '60岁及以上' OR '65岁及以上')

**7. Wanfang Data**

主题 = ('心力衰竭' OR '心衰' OR '慢性心力衰竭' OR '充血性心力衰竭' OR '急性心力衰竭' OR '心功能不全' OR '心脏衰竭' OR '心脏失代偿')
AND 主题 = ('认知衰弱' OR '认知虚弱' OR '认知衰弱症' OR '可逆性认知衰弱' OR '潜在可逆性认知衰弱' OR '认知' OR '认知功能' OR '认知障碍' OR '认知功能障碍' OR '认知功能下降' OR '认知下降' OR '认知减退' OR '轻度认知障碍' OR '主观认知下降')
AND 主题 = ('认知衰弱' OR '认知虚弱' OR '认知衰弱症' OR '衰弱' OR '虚弱' OR '老年衰弱' OR '身体衰弱' OR '躯体衰弱' OR '生理性衰弱' OR '衰弱综合征' OR '可逆性认知衰弱' OR '潜在可逆性认知衰弱')
AND 主题 = ('老年' OR '老年人' OR '老年患者' OR '高龄' OR '老龄' OR '中老年' OR '60岁及以上' OR '65岁及以上')

**8. VIP Database**

主题 = ('心力衰竭' OR '心衰' OR '慢性心力衰竭' OR '充血性心力衰竭' OR '急性心力衰竭' OR '心功能不全' OR '心脏衰竭' OR '心脏失代偿')
AND 主题 = ('认知衰弱' OR '认知虚弱' OR '认知衰弱症' OR '可逆性认知衰弱' OR '潜在可逆性认知衰弱' OR '认知' OR '认知功能' OR '认知障碍' OR '认知功能障碍' OR '认知功能下降' OR '认知下降' OR '认知减退' OR '轻度认知障碍' OR '主观认知下降')
AND 主题 = ('认知衰弱' OR '认知虚弱' OR '认知衰弱症' OR '衰弱' OR '虚弱' OR '老年衰弱' OR '身体衰弱' OR '躯体衰弱' OR '生理性衰弱' OR '衰弱综合征' OR '可逆性认知衰弱' OR '潜在可逆性认知衰弱')
AND 主题 = ('老年' OR '老年人' OR '老年患者' OR '高龄' OR '老龄' OR '中老年' OR '60岁及以上' OR '65岁及以上')

**9. SinoMed**

('心力衰竭'[主题词] OR '心力衰竭'[常用字段] OR '心衰'[常用字段] OR '慢性心力衰竭'[常用字段] OR '充血性心力衰竭'[常用字段] OR '急性心力衰竭'[常用字段] OR '心功能不全'[常用字段] OR '心脏衰竭'[常用字段] OR '心脏失代偿'[常用字段])
AND ('认知功能障碍'[主题词] OR '认知衰弱'[常用字段] OR '认知虚弱'[常用字段] OR '认知衰弱症'[常用字段] OR '可逆性认知衰弱'[常用字段] OR '潜在可逆性认知衰弱'[常用字段] OR '认知'[常用字段] OR '认知功能'[常用字段] OR '认知障碍'[常用字段] OR '认知功能障碍'[常用字段] OR '认知功能下降'[常用字段] OR '认知下降'[常用字段] OR '认知减退'[常用字段] OR '轻度认知障碍'[常用字段] OR '主观认知下降'[常用字段])
AND ('衰弱'[主题词] OR '认知衰弱'[常用字段] OR '认知虚弱'[常用字段] OR '认知衰弱症'[常用字段] OR '衰弱'[常用字段] OR '虚弱'[常用字段] OR '老年衰弱'[常用字段] OR '身体衰弱'[常用字段] OR '躯体衰弱'[常用字段] OR '生理性衰弱'[常用字段] OR '衰弱综合征'[常用字段] OR '可逆性认知衰弱'[常用字段] OR '潜在可逆性认知衰弱'[常用字段])
AND ('老年人'[主题词] OR '老年'[常用字段] OR '老年人'[常用字段] OR '老年患者'[常用字段] OR '高龄'[常用字段] OR '老龄'[常用字段] OR '中老年'[常用字段] OR '60岁及以上'[常用字段] OR '65岁及以上'[常用字段])
